# Supplementary material for: Metabolomics and Transcriptomics Reveal the Response Mechanisms of Mikania micrantha to Puccinia spegazzinii Infection
Source: Microorganisms. 2023 Mar 7;11(3):678. doi: 10.3390/microorganisms11030678 (PMC10057677; doi:10.3390/microorganisms11030678)
Supplement: Supplementary file 1 [file microorganisms-11-00678-s001.zip › Supplementary Figures.pdf]

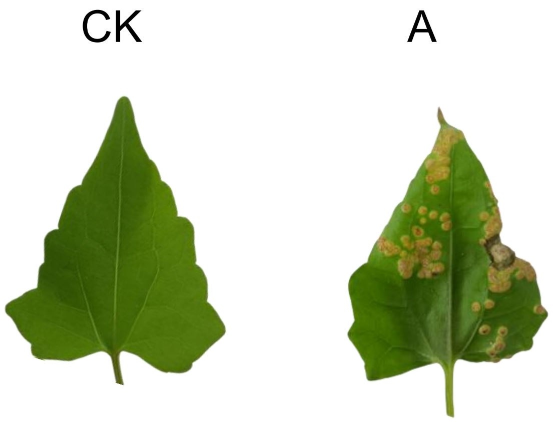

Supplementary Figure S1 Phenotypes of *P. spegazzinii* were observed and photographed 17 days after inoculation.

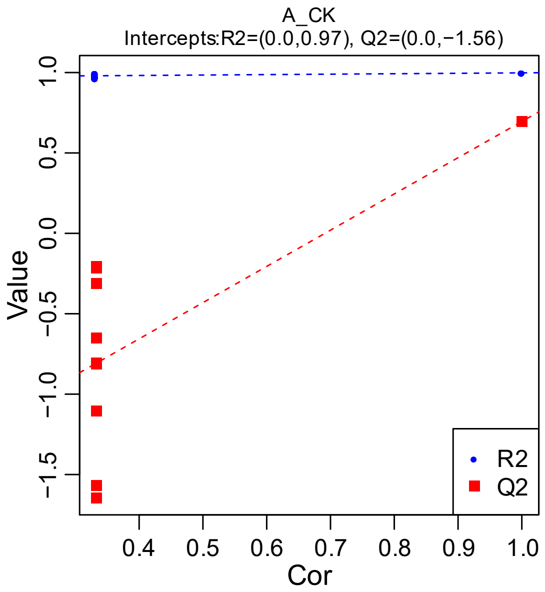

Supplementary Figure S2 Performance of the permutation tests validated from the PLS-DA model.

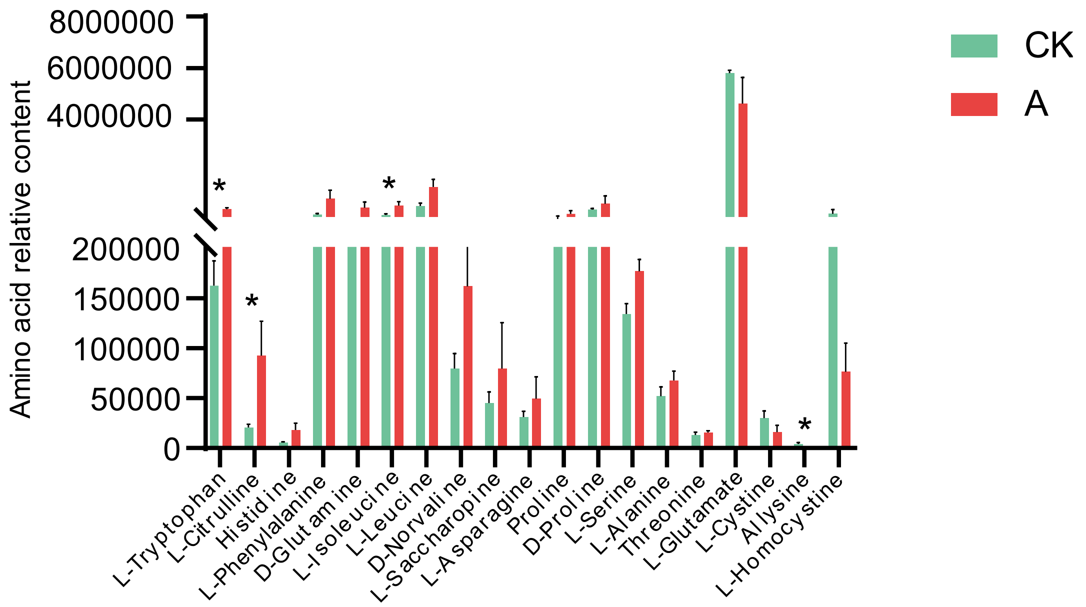

Supplementary Figure S3 The difference of amino acid contents in *M. micrantha* leaves between the uninfected and infected by *P. spegazzinii*.
